# Evidence summary for optimal glycemic variability management during enteral nutrition in adult ICU patients with cerebral infarction

**DOI:** 10.3389/fnut.2026.1822666

**Published:** 2026-05-19

**Authors:** Haishuang Mei, Chaxiang Li, Min Liu, Zhaohui Zhang, Chao Cao, Guilan Ban

**Affiliations:** 1 The First College of Clinical Medical Science, China Three Gorges University, Yichang Central People’s Hospital, Yichang, Hubei, China; 2 College of Health Sciences, China Three Gorges University, Yichang, Hubei, China

**Keywords:** best evidence, cerebral infarction, enteral nutrition, evidence-based nursing, glycemic variability, intensive care unit

## Abstract

**Objective:**

To systematically summarize the best available evidence on glycemic variability (GV) management during enteral nutrition (EN) in adult patients with cerebral infarction in the intensive care unit (ICU), and to provide an evidence-based reference for clinical practice.

**Methods:**

Guided by the 6S evidence-based model, this evidence summary systematically searched Chinese and English evidence-based resource databases, guideline websites, professional society websites, and literature databases from inception to April 1, 2026. Eligible documents were critically appraised using source-specific tools, and the best available evidence was extracted, graded, and synthesized.

**Results:**

A total of 18 documents were included, comprising 9 guidelines, 2 expert consensuses, 3 clinical decisions, 3 best evidence summaries and 1 systematic review. Thirty-five pieces of best evidence were summarized across five domains: basic patient assessment and screening, blood glucose monitoring and target control, optimization of enteral nutrition regimens, insulin intervention strategies, and multidisciplinary collaborative management.

**Conclusion:**

This study summarizes the best available evidence for GV management during EN in adult patients with cerebral infarction in the ICU. In clinical practice, individualized adjustments should be made according to patient conditions and local resource availability to improve the quality of GV management.

**Systematic review registration:**

This evidence summary has been registered at the Evidence Summary Registration Platform of Fudan University Centre for Evidence-Based Nursing. The registration details are as follows: Unique Identifier: ES20269922. Title: Evidence Summary of Glycemic Variability Management During Enteral Nutrition in Patients With Cerebral Infarction Registration date: 2026-02-13. The publicly accessible registration platform URL is: http://ebn.nursing.fudan.edu.cn/home.

## 1 Introduction

Cerebral infarction is the leading cause of death and disability among adults in China (1)^.^ Critically ill patients with cerebral infarction admitted to the ICU often present with dysphagia and metabolic disturbances due to severe illness and profound neurological deficits (2). EN, the preferred nutritional support modality for these patients, plays a pivotal role in improving nutritional status, facilitating neurological recovery, and shortening hospital stays (3). However, during EN, these patients are prone to elevated GV due to factors such as disease-related stress, reduced insulin sensitivity, fluctuations in nutritional infusion, and glucocorticoid administration (4). The adverse impact of GV on patient outcomes is far greater than that of isolated hyperglycemia or hypoglycemia; it can exacerbate neurological damage, increase the risk of complications such as infections and multiple organ dysfunction, and significantly impair long-term prognosis and quality of life (5).

Although existing stroke and inpatient management guidelines address general glycemic control, evidence directly relevant to glycemic variability management during enteral nutrition in adult patients with cerebral infarction in the ICU setting remains fragmented. In particular, clinically applicable recommendations regarding glucose fluctuation monitoring, EN optimization, insulin adjustment during feeding continuation or interruption, and multidisciplinary implementation are dispersed across various documents in stroke care, critical care, nutrition, and inpatient glycemic management. Therefore, this study aims to integrate the best available and clinically applicable evidence into an ICU-focused framework for glycemic variability management during enteral nutrition. Direct evidence specific to adult ICU patients with cerebral infarction receiving enteral nutrition remains limited; therefore, this review integrates both directly relevant evidence and cautiously selected indirect evidence from broader but clinically related populations.

## 2 Materials and methods

### 2.1 Formulation of the research question

The review question was formulated using the PICOS framework (6).

This study was registered in the Evidence-Based Nursing Center of Fudan University (Registration no. ES20232486).

### 2.2 Search strategy

The search was performed based on the “6S” pyramid evidence model (7) using a top-down approach. Preappraised evidence resources, guideline repositories/professional society websites, and bibliographic databases were searched systematically. The following databases and websites were searched: BMJ Best Practice, UpToDate, JBI Evidence-Based Health Care Center, American Society for Parenteral and Enteral Nutrition (ASPEN), European Society for Clinical Nutrition and Metabolism (ESPEN), American Stroke Association (ASA), Medlive, Society of Critical Care Medicine (SCCM), American College of Gastroenterology.

(ACG), CNKI, VIP, Wanfang Data, CBM, PubMed, Cochrane Library, Embase, and Web of Science. Database-specific search strategies were developed by combining controlled vocabulary (e.g., subject headings, where applicable) with free-text terms related to four core concepts: “Intensive Care Unit/Severe/seriously ill/Critical/critically ill”, “cerebral infarction/infarct, brain/infarction, cerebral/Stroke Cerebral Infarct/Ischemic stroke/Stroke/cerebral artery occlusion”, “Enteral nutrition/Nutrition, enteral/feeding, tube/Feeding, enteral/Tube feeding/gastric feeding/Enteral feeding/Nasogastric feeding”, and “Blood glucose/high blood glucose/elevated blood sugar/glycemic elevation/Glucose variability/Blood glucose fluctuation/blood glucose control/Blood sugar hyperglycemia”. The search range was from the inception of each database to April 1, 2026.

The eligibility language was limited to Chinese and English. Because guideline repositories and professional society websites often provide simple search interfaces and relevant guidance documents are frequently indexed under broader themes, a source-adapted search approach was used for these sources. When direct four-concept combined retrieval yielded very limited records, the core concepts were searched separately and iteratively, followed by eligibility screening of potentially relevant documents according to the predefined inclusion and exclusion criteria.

A full example search strategy for PubMed and the detailed search approach for guideline repositories are presented in Appendix.

### 2.3 Inclusion and exclusion criteria

Inclusion criteria: (1) Adult (≥18 years old) patients with cerebral infarction in the ICU receiving EN; (2) Study focus: interventions for the management of GV during EN; (3) Document types: clinical practice guidelines, clinical decisions, expert consensus statements, evidence summaries, and systematic reviews; (4) Language: Chinese and English.

Exclusion criteria: (1) Documents with incomplete basic information or unassessable quality; (2) Documents involving interpretation, translation, or commentary on existing guidelines or consensus; (3) outdated guidelines or consensus statements when updated versions were available; (4) Duplicate publications; (5) Documents for which full text was unavailable; and (6) pediatric studies.

### 2.4 Literature quality assessment

The methodological quality of clinical guidelines was appraised using the Appraisal of Guidelines for Research and Evaluation II (AGREE II) instrument (8). The instrument includes 6 domains and 23 items. Each item was scored on a scale from 1 to 7. The total score for each domain was then calculated and standardized as a percentage. The included systematic review was appraised using AMSTAR 2 (9). Expert consensus documents were appraised using the Joanna Briggs Institute (JBI) Critical Appraisal Tools for text and expert opinion papers (10). Evidence summaries and clinical decisions were appraised using the Critical Appraisal for Summaries of Evidence (CASE) tool (11). Only documents meeting the prespecified methodological standards for their source type were retained for synthesis, and the appraisal results were subsequently considered during evidence prioritization and interpretation (Table 1).

**Table 1:** Research methods for the evidence summary: PICOS framework, search strategy, inclusion/exclusion criteria, and quality appraisal tools.

Item	Description
Criteria for summarizing evidence	P (Population): adult patients with cerebral infarction in the ICU receiving enteral nutrition; I (Intervention): strategies for glycemic variability management during enteral nutrition; C (Comparator): conventional glycemic management strategies, where applicable; O (Outcomes): glycemic variability and clinical prognosis; S (Study design): clinical practice guidelines, clinical decisions, expert consensus statements, evidence summaries, and systematic reviews.
Inclusion criteria	(i) Studies involving adult patients with cerebral infarction in the ICU; (ii) Studies focusing on measures for managing glycemic fluctuations during EN; (iii) Evidence types including clinical practice guidelines, clinical decision recommendations, expert consensuses, evidence summaries, and systematic reviews; (iv) Publications in Chinese or English.
Exclusion criteria	(i) Studies with incomplete basic information or unassessable quality; (ii) Interpretations, translations, or commentaries on existing guidelines or consensuses; (iii) Updated versions of previously published guidelines or consensuses; (iv) Duplicate publications; (v) Studies for which full text was unavailable.
Evidence-based information databases/platforms	BMJ Best Practice, UpToDate, JBI Evidence-Based Health Care Centre website, American Society for Parenteral and Enteral Nutrition (ASPEN), European Society for Clinical Nutrition and Metabolism (ESPEN), American Stroke Association (ASA), Medlive Guideline Network, Society of Critical Care Medicine (SCCM), American College of Gastroenterology (ACG).
Other comprehensive databases	China National Knowledge Infrastructure (CNKI), VIP Chinese Journal Database, Wanfang Data, China Biology Medicine disc (CBM), PubMed, Cochrane Library, Embase, Web of Science.
Chinese and English search keywords	Database-specific search strategies were developed using controlled vocabulary and free-text terms for intensive care/critical illness, cerebral infarction/ischemic stroke, enteral nutrition, and glycemic variability/blood glucose management. Full search strings for a major database are provided in Appendix.
Grey literature and guideline repositories	Guideline repositories and professional society websites were searched systematically using the same core concepts, and current or most recently updated relevant documents were screened.
Search period	From database establishment to April 1, 2026.
Tool for quality evaluation of guidelines	Appraisal of Guidelines for Research and Evaluation II (AGREE II) (8)
Tool for quality evaluation of expert consensuses	Joanna Briggs Institute (JBI) Critical Appraisal Tools for text and expert opinion papers (10)
Tool for quality evaluation of evidence summaries/clinical decisions	Critical Appraisal for Summaries of Evidence (CASE) (11)
Tool for evidence grading	2014 version of the JBI Pre-grading System for Evidence (13)
Tool for Systematic Review	A measurement Tool to Assess Systematic Reviews 2 (AMSTAR 2) (9)

### 2.5 Evidence extraction and synthesis

Two investigators with evidence-based methodology training independently assessed and comprehensively reviewed all items to determine document inclusion. When consensus could not be reached, a third investigator was consulted to resolve discrepancies and reach a final decision. Principles for evidence synthesis (12) were as follows: (1) the included sources were not treated as equivalent during synthesis; clinical practice guidelines and the systematic review were prioritized as principal evidence sources, expert consensus documents were considered supportive evidence, and clinical decisions/evidence summaries were used as supplementary secondary sources; (2) for consistent evidence, higher-quality and methodologically stronger evidence was preferentially retained; (3) for complementary evidence, items were logically combined to form integrated evidence statements, and secondary sources were used to refine implementation details without overriding higher-level evidence; (4) For conflicting evidence priority was given to high-quality, recently published, and locally applicable evidence (13); and (5) when direct evidence from adult ICU patients with cerebral infarction receiving enteral nutrition was available, it was prioritized, whereas indirect evidence from broader stroke, critical care, or inpatient populations was included only when directly relevant to the review question and interpreted cautiously.

## 3 Results

### 3.1 Literature search results

**Figure 1:**
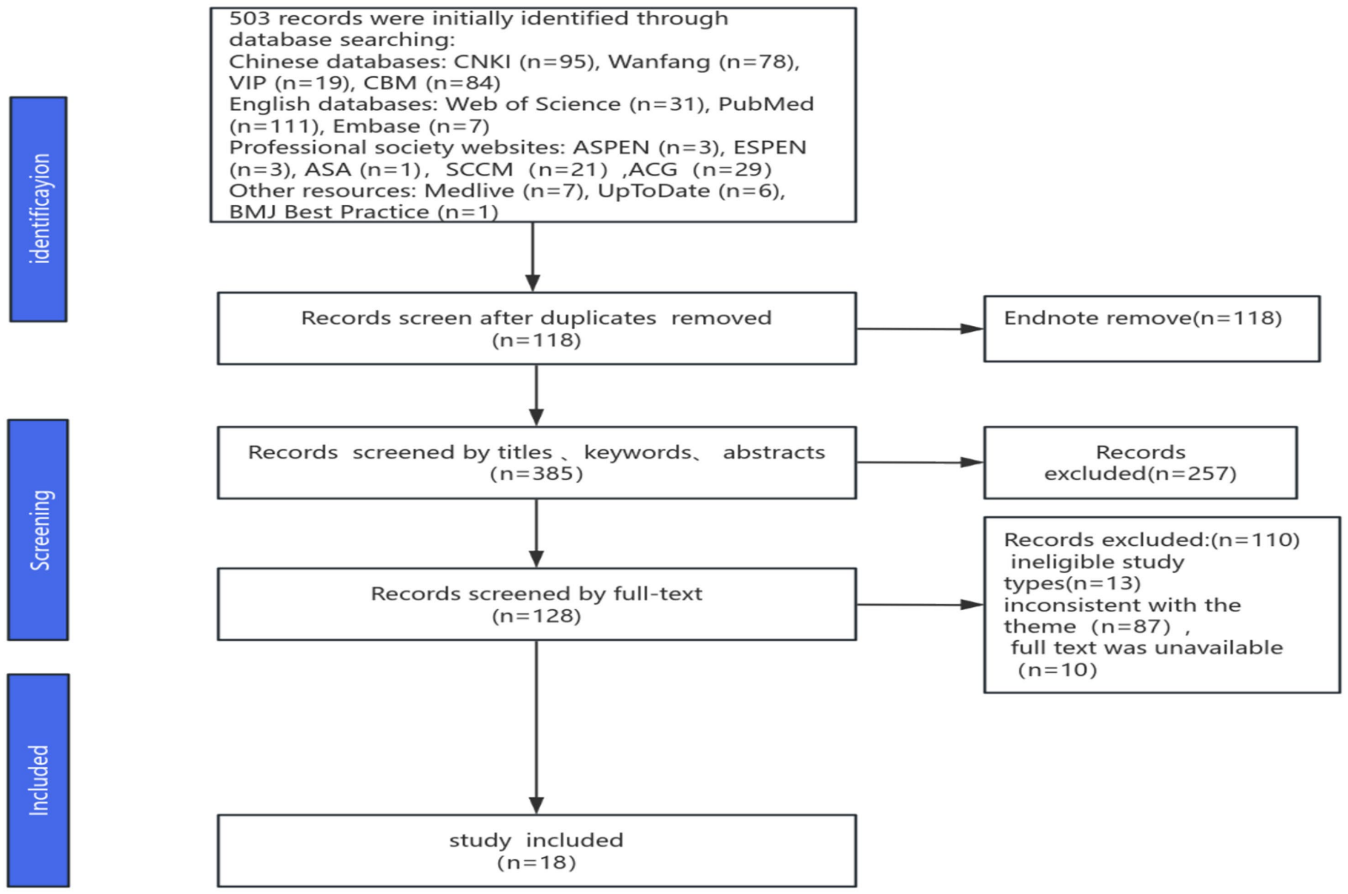
Literature screening flowchart and results.

A total of 503 records were initially retrieved. After 118 duplicate records were removed using EndNote, 257 records were excluded after title and abstract screening. A further 128 records were excluded after full-text review. Finally, 18 documents were included, comprising 9 clinical guidelines (14–22), 2 expert consensus statements (23, 24), 3 clinical decision tools (25–27), 3 best evidence summaries (28–30), and 1 systematic review (31). The literature screening process is illustrated in Figure 1 and the general characteristics of the included documents are presented in Table 2.

**Table 2:** General characteristics of the included documents (*n =* 18).

Included documents	Source	Type of evidence	Topic	Year
Wu et al. (14)	VIP	Clinical guideline	Chinese Guidelines for the Management of Severe Stroke 2024	2024
Compher et al. (15)	ASPEN	Clinical guideline	Guidelines for the provision of nutrition support therapy in the adult critically ill patient: The American Society for Parenteral and enteral nutrition	2022
Singer et al. (16)	ESPEN	Clinical guideline	ESPEN guideline on clinical nutrition in the intensive care unit	2019
Wunderle et al. (17)	ESPEN	Clinical guideline	ESPEN guideline on nutritional support for polymorbid medical inpatients	2023
National Health Commission of the People’s Republic of China. (18)	Medlive	Clinical guideline	Chinese Guidelines for the Prevention and Treatment of Stroke	2021
West China evidence-based nursing Center, Sichuan University, Nursing Management Committee, Chinese nursing association, neurosurgery branch, Chinese Medical Association (19)	Medlive	Clinical guideline	Chinese Guidelines for enteral nutrition Care in Stroke	2021
Roberts et al. (20)	Medlive	Clinical guideline	Glycaemic management during the inpatient enteral feeding of stroke patients with diabetes	2018
Joint British Diabetes Societies for Inpatient Care (JBDS-IP) group (21)	Diabetes UK	Clinical guideline	Glycaemic management during enteral feeding for people with diabetes in hospital	2024
Honarmand et al. (22)	SCCM	Clinical guideline	Society of Critical Care Medicine Guidelines on Glycemic Control for Critically Ill Children and Adults 2024	2024
Wu et al. (23)	PubMed	Expert consensus	Expert consensus on the glycemic management of critically ill patients	2022
Rebollo-Perez MI et al. (24)	PubMed	Expert consensus	Standards for the Use of enteral nutrition in Patients with Diabetes or Stress Hyperglycaemia: Expert Consensus	2023
Heuschkel et al. (25)	UpToDate	Clinical decision	Enteral nutrition: Gastric Feeding and Postpyloric Feeding	2025
Oliveira-Filho et al. (26)	UpToDate	Clinical decision	Primary Assessment and Management of Acute Stroke	2026
Seres et al. (27)	UpToDate	Clinical decision	Nutritional Support for Critically Ill Adults: Parenteral Nutrition	2026
Xu et al. (28)	CNKI	Best evidence summary	Best evidence summary for the management of intravenous insulin infusion in ICU patients	2023
Shi et al. (29)	CNKI	Best evidence summary	Best evidence summary for the management of hyperglycemia after enteral nutrition in patients with severe stroke	2024
Wang et al. (30)	CNKI	Best evidence summary	Best evidence summary for the management of hyperglycemia during enteral nutrition in critically ill patients	2022
Hryciw et al. (31)	SCCM	Systematic Review	Glycemic Variability As a Prognostic Factor for Mortality in Patients With Critical Illness: A Systematic Review and Meta-Analysis	2024

### 3.2 Quality assessment of included documents

#### 3.2.1 Clinical guidelines

The appraisal results of the 9 guidelines are presented in Table 3. Among them, 6 guidelines were graded as Grade A (14–18, 22), and the remaining 3 as Grade B (19–21). The inter-rater reliability between the two researchers was satisfactory, and all guidelines were included in the analysis.

**Table 3:** Methodological quality assessment results of the included guidelines.

Included guidelines	Standardized domain scores (%)						Number of domains ≥60%
	Scope and purpose	Stakeholder involvement	Rigor of development	Clarity and presentation	Applicability	Editorial independence	
Wu et al. (14)	97.2	69.4	65.7	60.4	62.5	91.7	6
Compher et al. (15)	100	66.7	89.8	83.3	29.2	87.5	5
Singer et al. (16)	94.4	66.7	98.1	81.3	66.7	91.7	6
Wunderle et al. (17)	100.0	72.2	98.1	83.3	83.3	100.0	6
Liu et al. (18)	95.2	76.2	68.6	80.0	68.4	61.9	6
West China evidence-based nursing Center, Sichuan University, Nursing Management Committee, Chinese nursing association, neurosurgery branch, Chinese Medical Association (19)	95.2	88.1	98.1	91.1	95.2	85.7	6
Roberts et al. (20)	100.0	76.2	59.3	89.3	73.8	71.4	6
Joint British Diabetes Societies for Inpatient Care (JBDS-IP) group (21)	100.0	80.9	79.8	94.6	81.0	71.4	6
Honarmand et al. (22)	98.4	79.6	78.8	92.4	78.6	71.2	6

#### 3.2.2 Expert consensus

A total of 2 expert consensus documents were included in this study (23, 24). According to the appraisal criteria, all items were rated “yes” except for the item “whether any inconsistent views with previous literature were identified”, which was rated “unclear.” The overall quality was high, and both documents were included.

#### 3.2.3 Clinical decisions and evidence summaries

Three clinical decision tools from UpToDate were included (25–27), which were treated as supplementary secondary evidence and were used mainly to support interpretation and implementation details rather than to outweigh higher-level evidence. Three evidence summaries were retrieved from CNKI (28–30). Among them, 2 summaries (28, 29) were rated “yes” for all items except “assessment of publication bias”, which was rated “no”; these were deemed high quality and included. All items of 1 summary (30) were rated “yes”, indicating high overall quality, and it was included.

#### 3.2.4 Systematic reviews

The quality assessment results of the systematic review are presented in Table 4.

**Table 4:** Quality assessment results of systematic reviews (*n =* 1).

Included literature	①	②	③	④	⑤	⑥	⑦	⑧	⑨	⑩	⑪
Hryciw et al. (31)	Yes	Yes	Yes	Yes	Yes	Yes	Yes	Yes	Yes	No	Yes

① Whether the evidence-based question was clearly and explicitly stated; ② Whether the eligibility criteria for literature were appropriate; ③ Whether the search strategy was adequately designed and implemented; ④ Whether the sources of included primary studies were appropriate; ⑤ Whether the tool used for quality assessment was appropriate; ⑥ Whether quality assessment was conducted independently by at least two reviewers; ⑦ Whether measures were taken to reduce errors during data extraction; ⑧ Whether the methods for synthesizing and/or pooling studies were appropriate; ⑨ Whether publication bias was appropriately assessed; ⑩ Whether recommendations for policy or practice were supported by the reported data; ⑪ Whether appropriate suggestions for specific directions of future research were provided.

### 3.3 Evidence grading and synthesis

Finally, 35 pieces of best evidence were synthesized from 5 domains: basic assessment and screening, monitoring of blood glucose fluctuations, optimization of EN regimens, insulin intervention strategies, and multidisciplinary collaborative management, as shown in Table 5.

**Table 5:** Summary of best evidence for GV management during EN in adult ICU patients with cerebral infarction.

Evidence topic	Evidence content	Level
Basic patient assessment and screening	1. Nutritional risk screening is recommended using the Nutritional Risk Screening 2002 (NRS2002) for stroke patients; the Mini Nutritional Assessment Short Form is preferred for elderly patients (15, 16, 19).	1b
	2. EN support is recommended to be initiated within 48 h of admission for stroke patients at high nutritional risk (NRS2002 ≥ 3) (18, 19).	1a
	3. Glycated hemoglobin (HbA1c) should be routinely measured upon ICU admission (23).	2b
	4. Patients with diabetes, elevated HbA1c, or hyperglycemia with metabolic stress are at high risk of blood glucose fluctuations (21, 24).	3b
	5. Swallowing function in stroke patients should be screened using the Global Bedside Evaluation of Swallowing after Stroke (GLOBE-3S) or Bedside Clinical Assessment (BCA) (30).	3a
	6. The feasibility of EN should be comprehensively determined based on swallowing function screening and gastrointestinal tolerance (19).	3a
Blood glucose monitoring and target control	7. Monitoring the glucose coefficient of variation (GLUcv) is recommended for critically ill patients; it reflects the amplitude of GV and is calculated as (standard deviation of blood glucose ×100)/mean blood glucose (23, 31).	3a
	8. Priority order for blood glucose sampling: arterial blood > venous blood > capillary blood (23, 26, 27).	3b
	9. Venous blood sampling should be avoided from the ipsilateral arm of glucose-containing infusions (26).	4a
	10. In patients with limb dysfunction (e.g., hemiplegia), capillary blood sampling is recommended from the unaffected extremity (27).	3b
	11. Continuous Glucose Monitoring (CGM) may be used in patients with large GV to provide dynamic trend data (23).	3b
	12. In newly admitted ICU patients or those with unstable insulin infusion, blood glucose monitoring interval should be ≤1 h until blood glucose and insulin rate are stable (20, 21, 25, 27).	3b
	13. After stable blood glucose for 12–24 h and stable insulin infusion, monitoring may be extended to every 4 h (20, 21, 23, 27).	5b
	14. Glycemic target for cerebral infarction patients: 7.8–10.0 mmol/L (14, 22, 23, 26).	5b
	15. Glycemic target may be relaxed to 6.1–11.1 mmol/L for stroke patients with diabetes (23, 24).	3a
Optimization of EN regimen	16. Formula optimization: diabetes-specific formula is preferred for patients with diabetes or stress hyperglycemia (23, 29, 30)	1a
	17. Glucose or carbohydrate intake should not exceed 5 mg/kg/min in enteral or parenteral nutrition (16, 23)	5b
	18. Enteral access: nasogastric tube for short-term use (<4 weeks); percutaneous endoscopic gastrostomy for long-term use (>4 weeks) (18, 25, 30).	3b
	19. Post-pyloric feeding may be considered for patients at high risk of aspiration (e.g., gastroparesis, impaired consciousness) (16, 17, 24–26).	5b
	20. Continuous EN plus subcutaneous insulin is preferred for patients with large glycemic fluctuations (21, 26).	5b
	21. Insulin dosage should be adjusted synchronously (usually increased by 10–20%) when increasing the feeding rate (21).	5b
	22. Enteral formula should be warmed to 37–40 °C to improve tolerance (19, 29).	1a
	23. EN should be paused gradually: reduce infusion rate by half for 1–2 h before complete cessation (21, 30).	5b
Insulin intervention strategy	24. Insulin initiation threshold: ≥10 mmol/L during EN (14, 16, 18, 22, 23, 26, 29).	3b
	25. Short-acting insulin is preferred for glycemic management (14, 17, 20, 21, 23, 24, 28).	5b
	26. Intravenous insulin infusion is recommended in the acute phase (18, 21, 22, 29).	5b
	27. Intravenous insulin preparation: concentration 1 U/mL; flush the line with 20 mL solution before infusion to reduce adsorption (22, 27–29).	5b
	28. Initial intravenous insulin infusion rate: 1–2 U/h, titrated every 1–2 h (21–23, 28–30).	5b
	29. For patients on continuous EN, combination of basal insulin + rapid-acting prandial insulin is recommended (21, 23, 30).	5b
	30. Dosage adjustment: increase by 2–4 U or decrease by 1–2 U per step according to GV (20, 21).	5b
	31. Monitor blood glucose 1 h before interruption of EN; if 7.8–10.0 mmol/L, reduce insulin infusion rate by 20–30% first (21–23, 30).	5b
	32. Discontinue insulin immediately when blood glucose <3.9 mmol/L (28).	1a
	33. For hypoglycemia: administer 15–20 g of 50% glucose intravenously immediately (14, 22, 28–30)	1a
	34. High-risk hypoglycemia patients: monitor every 1–2 h; after hypoglycemia treatment, monitor within 15 min until stable in the target range (23).	5b
Multidisciplinary collaborative management	35. A multidisciplinary team is recommended for nutrition management, including urologists, specialized nurses, dietitians, rehabilitation therapists, and psychologists (19–21, 23).	1a

**Figure 2:**
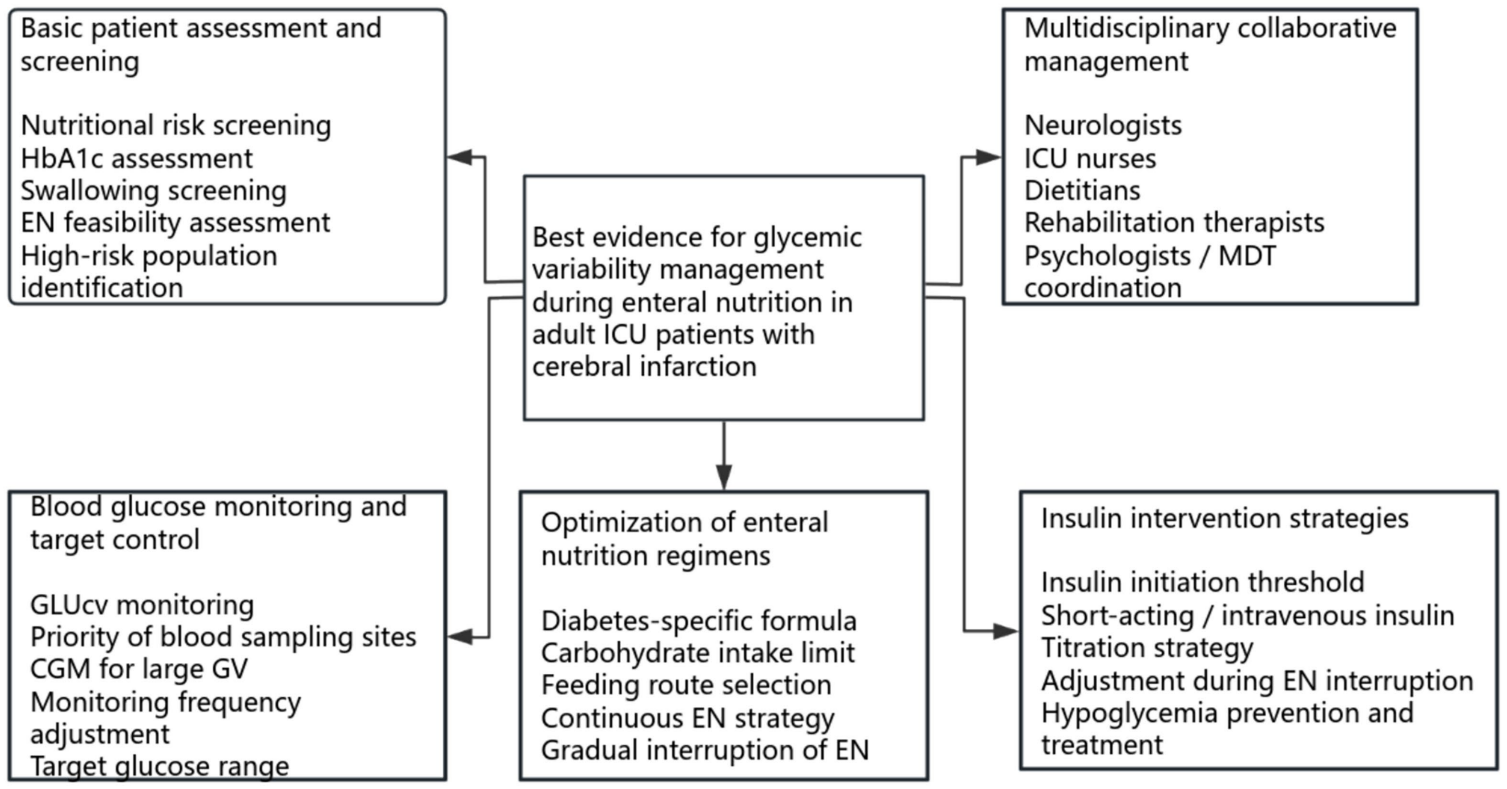
Best evidence for GV management during EN in adult ICU patients with cerebral infarction. GV, glycemic variability; EN, enteral nutrition; HbA1c, glycated hemoglobin; GLUcv, glucose coefficient of variation; CGM, continuous glucose monitoring; MDT, multidisciplinary team.

**Figure 3:**
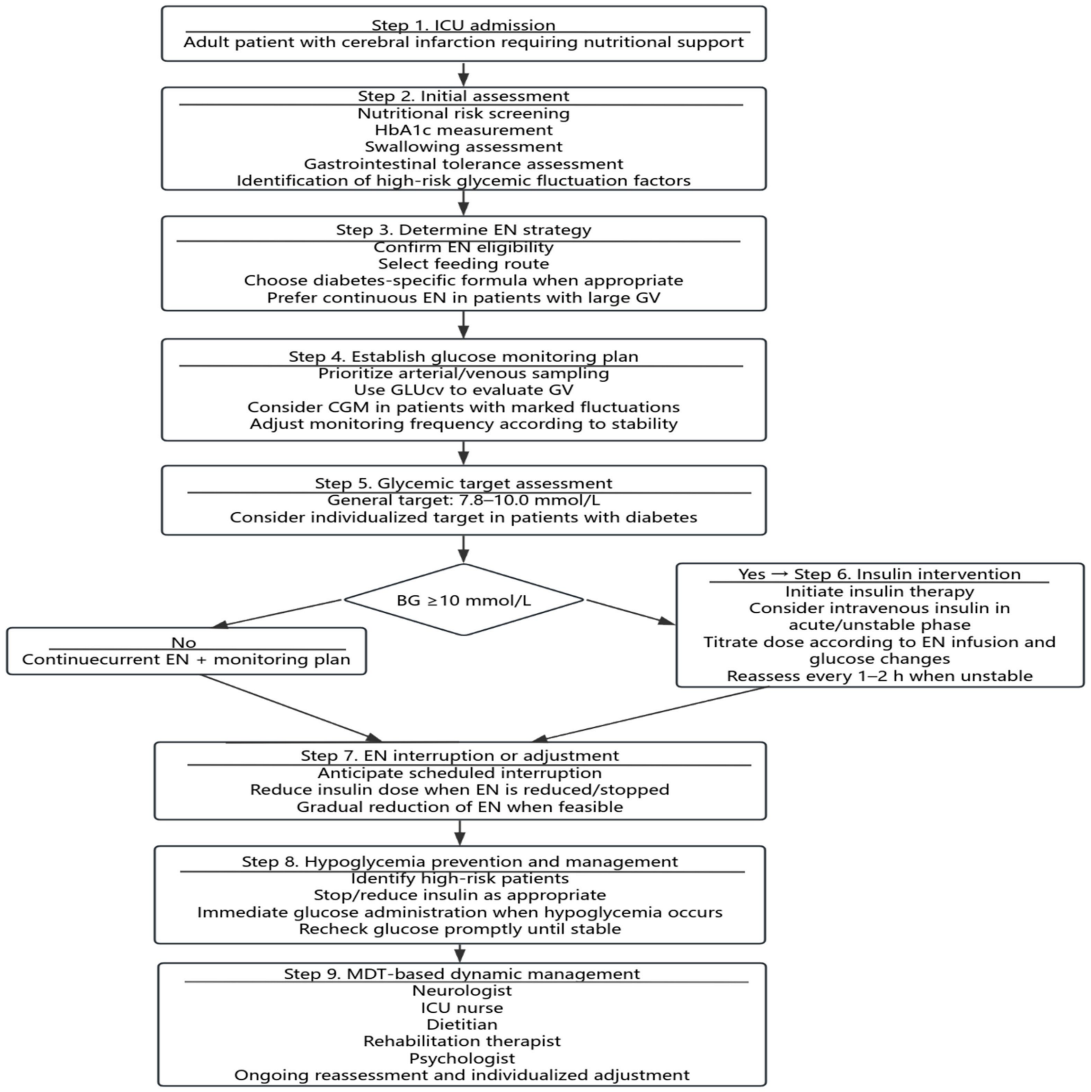
Evidence-informed clinical pathway for GV management during EN in adult ICU patients with cerebral infarction. This figure was derived from the synthesized evidence statements included in this review and is intended to support clinical interpretation; it has not undergone separate external validation. BG, blood glucose; GV, glycemic variability; EN, enteral nutrition; HbA1c, glycated hemoglobin; GLUcv, glucose coefficient of variation; CGM, continuous glucose monitoring; MDT, multidisciplinary team.

To enhance the visual synthesis and clinical applicability of the findings, the 35 evidence statements were further reorganized into two graphical presentations. Figure 2 summarizes the overall framework of the synthesized evidence across five domains, whereas Figure 3 was derived from these synthesized evidence statements and reorganized into a stepwise evidence-informed clinical pathway covering assessment, monitoring, enteral nutrition management, insulin intervention, and multidisciplinary collaboration.

## 4 Discussion

Overall, the synthesized evidence showed broad agreement regarding the need for early nutritional risk assessment, structured blood glucose monitoring, individualized glycemic targets, optimization of enteral nutrition delivery, and multidisciplinary collaboration. However, uncertainty remains regarding subgroup-specific glucose targets, the routine interpretation and application of glycemic variability metrics, and the standardization of insulin titration and interruption strategies in this highly specific ICU population.

### 4.1 Basic patient assessment and screening

Evidence from this study indicates that nutritional risk screening is not only a prerequisite for initiating EN (15, 16, 19), but also an independent risk factor for predicting elevated GV (32). Patients with an NRS2002 score ≥3 should initiate EN within 48 h. Evidence-based rationale is that early nutritional support maintains intestinal mucosal barrier integrity, reduces bacterial translocation and systemic inflammation, thereby alleviating stress-induced insulin resistance (IR) and patho-physiologically lowering the risk of elevated GV (33, 34). Nevertheless, the relationship between early EN initiation and subsequent GV improvement in this specific ICU cerebral infarction population remains supported mainly by indirect physiological and critical care evidence, rather than by direct comparative studies specifically focused on GV.

Cerebral infarction patients are often complicated by brain-gut axis dysfunction. Such dysregulation of the neuro-endocrine-immune network further exacerbates abnormal glucose metabolism, suggesting that nutritional risk assessment should incorporate multidimensional indicators such as neurological deficit severity and stress level to establish a comprehensive risk evaluation system (35). This broader multidimensional approach appears clinically reasonable, but its operationalization and weighting in routine ICU practice have not yet been standardized.

Baseline measurement of glycated hemoglobin (HbA1c) has dual clinical value: first, it effectively differentiates stress hyperglycemia from pre-diabetes and clarifies the pathological basis of IR (23). Second, it provides an individualized reference threshold for subsequent GV monitoring. Patients with high HbA1c have pre-existing chronic hyperglycemia-induced *β*-cell dysfunction and are more prone to glucose fluctuations under the carbohydrate load of EN (36). Clinically, a graded early warning system should be established at the risk assessment stage to implement individualized threshold management, replacing the traditional extensive single-threshold management mode. However, although the rationale for incorporating HbA1c into early risk stratification is strong, current evidence remains insufficient to support a fully standardized graded early-warning model for this specific clinical scenario.

### 4.2 Precision blood glucose monitoring

Different from the traditional concept of blood glucose management that focuses on absolute glycemic control, this evidence-based summary highlights the clinical value of GV as an independent prognostic predictor in ICU patients with cerebral infarction (23). The glucose coefficient of variation integrates two key dimensions, namely amplitude and frequency of glycemic fluctuations, to reflect glucose metabolic stability more comprehensively and objectively, thus overcoming the limitations of traditional single-point blood glucose monitoring (37). At the same time, the clinical interpretation of GV metrics in routine practice remains incompletely standardized, and the threshold at which GV should trigger escalation of management has not been clearly established.

For monitoring techniques, arterial blood sampling is prioritized for glucose measurement, based on evidence that different blood sources introduce systematic errors in finger-prick glucose testing. Cerebral infarction patients often present with peripheral circulatory disorders such as hemiplegia, which significantly widens the glucose concentration difference among capillary, venous, and arterial blood (23, 25, 26). Exclusive reliance on capillary blood monitoring may lead to overestimation or underestimation of GV, compromising the accuracy of clinical decision-making (38).

The “intensive monitoring followed by gradual extension” strategy recommended in this study represents a practical application of risk-adaptive management in GV control. This strategy not only avoids resource waste from excessive medical intervention but also captures glycemic fluctuations promptly during critical periods of insulin titration, ensuring patient safety. Notably, the target glucose range (6.1–11.1 mmol/L) is more lenient for patients with diabetes than for those without diabetes (22, 28). This is because strict glycemic control increases the risk of hypoglycemia, which may worsen patient outcomes. A balance between lenient control and neurological protection is required in clinical practice. The brain tissue may have a lower tolerance threshold for hyperglycemia under acute cerebral ischemia, and excessively loose glycemic control may aggravate ischemic brain injury (39). Across sources, there was broad agreement on the need for intensive early monitoring and the preference for more reliable sampling sources; however, uncertainty persists regarding the optimal target range for specific subgroups and the role of CGM in routine implementation.

### 4.3 Optimization of EN regimens

The design of EN regimens should be based on the precise matching between nutrient metabolic kinetics and the time-action curve of insulin. Diabetes-specific formulas, formulated with low-glycemic-index carbohydrates and monounsaturated fats, can delay gastric emptying and glucose absorption, thereby reducing the amplitude between postprandial glucose peaks and troughs (23, 29, 30). However, the feasibility of the evidence-based glucose intake limit of ≤5 mg/kg/min in the acute hypermetabolic state should be individually balanced against dual energy-protein targets to avoid compromising nutritional adequacy for the sake of GV control (16, 23). This reflects an important clinical tension: although carbohydrate restriction may theoretically reduce GV, excessive restriction may also increase the risk of underfeeding in metabolically stressed patients.

The selection of feeding route should be based on pathophysiological considerations of aspiration risk and delayed gastric emptying. Although post-pyloric feeding reduces gastric retention-related complications, it bypasses the gastric glucose-sensing mechanism, which may impair the fine neuro-humoral regulation of blood glucose and paradoxically increase GV (40). This suggests that route selection should be a graded decision based on the severity of gastroparesis, rather than a one-size-fits-all approach.

In addition, surgical and radiologic placement of enteral access tubes, such as percutaneous endoscopic gastrostomy (PEG) and radiologically inserted gastrostomy (RIG), should be fully considered for patients requiring long-term EN support. These techniques provide more stable and durable enteral access, reduce the risk of catheter displacement or interruption of feeding, and further contribute to maintaining stable glycemic control. Rouhi et al. demonstrated that radiologic gastrostomy was associated with better perioperative outcomes than surgical gastrostomy among the stroke patients with dysphagia (41), which is particularly important for continuous EN and GV control in critically ill patients with cerebral infarction. Furthermore, controlling EN infusion temperature and adopting a stepwise reduction strategy during interruption essentially provides physiological buffering of GV by maintaining the homeostasis of gastrointestinal hormone secretion and avoiding glucose “crash” event (42). In this domain, evidence was relatively consistent in supporting formula optimization and avoidance of excessive carbohydrate load, whereas recommendations on enteral route selection, warming, and interruption procedures were more dependent on indirect evidence and clinical context.

### 4.4 Insulin intervention strategies

Insulin therapy is the core intervention for reducing GV, but evidence indicates that its application should be based on a dynamic titration model grounded in the continuity of EN infusion. Intravenous insulin infusion, with its short half-life and rapid dose titration, is suitable for patients with severe glycemic fluctuations during the initiation phase of EN (43). However, the evidence-based protocol of an initial rate of 1–2 U/h with titration every 1–2 h (21, 24, 27) lacks individualization for varying degrees of insulin resistance. In clinical practice, it is recommended to calibrate the initial dose according to body mass index and inflammatory marker levels. Although such individualized dose adjustment appears clinically reasonable, the current evidence base does not yet provide sufficiently standardized criteria for its implementation.

Prophylactic insulin reduction before EN interruption is a key step in preventing iatrogenic hypoglycemia, but current evidence does not clearly distinguish between strategies for scheduled and unscheduled interruptions. Studies have confirmed that strict GV control may increase the incidence of nocturnal asymptomatic hypoglycemia. Patients with cerebral infarction exhibit blunted hypoglycemia awareness due to autonomic dysfunction, and the combination of these two factors significantly increases patient safety risk (44). Thus, although the available evidence broadly supports the importance of insulin-based management and close glucose monitoring, uncertainty remains regarding the standardization of dose initiation, transition, and interruption management for adult ICU patients with cerebral infarction receiving enteral nutrition.

### 4.5 Multidisciplinary collaborative management

GV management involves multiple disciplines including nutritional support, glycemic control, and neurological monitoring. The establishment of a multidisciplinary team (MDT) provides a structural guarantee for the implementation of evidence-based practice (18–20, 22). However, current evidence provides vague descriptions of the specific operational mechanism of the MDT. The MDT recommended in this study includes psychological counselors, reflecting the “physio-psychological” dual-management concept and aligning with the developmental trend of modern critical care medicine. In clinical practice, a decision support system based on electronic health records should be established to achieve closed-loop adjustment of EN infusion rate and insulin dosage.

In summary, the value of the present evidence summary lies not in redefining general glycemic control in stroke, but in integrating evidence specifically relevant to glycemic variability management during enteral nutrition in adult ICU patients with cerebral infarction into a more focused clinical framework. At the same time, the controversies highlighted above—such as subgroup-specific glycemic targets, the routine applicability of GV metrics, and the standardization of insulin interruption strategies—reflect genuine limitations of the current evidence base rather than fully resolved clinical standards.

## 5 Conclusion

This study summarized 35 pieces of the best evidence for glycemic variability management during enteral nutrition in adult ICU patients with cerebral infarction using evidence-based methods. The evidence covered five domains: basic patient assessment and screening, blood glucose monitoring and target control, optimization of enteral nutrition regimens, insulin intervention strategies, and multidisciplinary collaborative management. It provides an evidence-based reference for clinical staff involved in the management of this patient population. In clinical practice, individualized adjustments should be made according to patient conditions and local diagnostic and treatment resources to achieve more precise glycemic management.

### 5.1 Limitations of the review

Several limitations of this evidence summary should be acknowledged. First, this review was restricted to adult ICU patients with cerebral infarction receiving enteral nutrition; therefore, pediatric evidence was outside the scope of this review, and the findings should not be generalized to younger populations. Second, not all included source documents were developed exclusively for adult ICU patients with cerebral infarction receiving enteral nutrition; some were derived from broader stroke, critically ill, or inpatient populations. Accordingly, part of the supporting evidence remains indirect, and the external validity of some recommendations may be context-dependent and should be interpreted with caution. Third, notable heterogeneity existed across the included studies in terms of study design, baseline patient characteristics, and glycemic monitoring protocols, which precluded quantitative meta-analysis. Therefore, the conclusions were based on qualitative evidence synthesis and may retain some degree of subjectivity. Finally, the evidence included in this review was limited to publications available up to April 1, 2026, and future updates will be required as new high-quality evidence emerges. In addition, although Figure 3 was developed by reorganizing the synthesized evidence into a clinically oriented pathway, it has not undergone separate external validation or a dedicated *post-hoc* expert consensus procedure, and therefore should be interpreted as an evidence-informed support tool rather than a formally validated protocol.

## Data Availability

The original contributions presented in the study are included in the article/supplementary material, further inquiries can be directed to the corresponding author.

## Appendix

Detailed search strategy

Database: PubMed.

Search date: April 11, 2026.

Time limit: From database inception to April 1, 2026.

Language restriction: English and Chinese.

Filters: No methodological filters were applied. Controlled vocabulary terms were searched using MeSH headings, and free-text terms were searched in All Fields.

#1 Intensive care / critical illness.

(“Intensive Care Units”[Mesh] OR “Intensive Care Unit”[All Fields] OR “ICU”[All Fields] OR “Severe”[All Fields] OR “seriously ill”[All Fields] OR “Critical”[All Fields] OR “critically ill”[All Fields])

#2 Cerebral infarction / ischemic stroke.

(“Cerebral Infarction”[Mesh] OR “infarct, brain”[All Fields] OR “infarction, cerebral”[All Fields] OR “Stroke Cerebral Infarct”[All Fields] OR “Ischemic stroke”[All Fields] OR “Stroke”[All Fields] OR “cerebral artery occlusion”[All Fields] OR “brain infarction”[All Fields] OR “cerebral ischemic infarction”[All Fields] OR “ischemic cerebral infarction”[All Fields] OR “cerebral infarct”[All Fields] OR “brain ischemic stroke”[All Fields] OR “cerebral artery thrombosis”[All Fields])

#3 Enteral nutrition.

(“Enteral Nutrition”[Mesh] OR “Nutrition, enteral”[All Fields] OR “feeding, tube”[All Fields] OR “Feeding, enteral”[All Fields] OR “Tube feeding”[All Fields] OR “gastric feeding”[All Fields] OR “Enteral feeding”[All Fields] OR “Nasogastric feeding”[All Fields] OR “EN”[All Fields] OR “enteral alimentation”[All Fields] OR “enteral nutritional support”[All Fields] OR “enteral nutritional therapy”[All Fields] OR “post-pyloric feeding”[All Fields] OR “nasojejunal”[All Fields] OR “feeding”[All Fields] OR “feeding via tube”[All Fields] OR “tube-feeding”[All Fields] OR “artificial enteral feeding”[All Fields])

4 Blood glucose / glycemic variability.

(“Blood Glucose”[Mesh] OR “Glucose variability”[All Fields] OR “Blood glucose fluctuation”[All Fields] OR “blood glucose control”[All Fields] OR “Blood sugar”[All Fields] OR “high blood glucose”[All Fields] OR “elevated blood sugar”[All Fields] OR “glycemic elevation”[All Fields] OR “hyperglycemia”[All Fields] OR “Blood glucose management”[All Fields] OR “Hypoglycemia”[All Fields] OR “glucose profile”[All Fields] OR “blood glucose level”[All Fields])

#5 Final search.

#1 AND #2 AND #3 AND #4.

## References

[1] Watkins DA. Policy priorities for preventing stroke-related mortality and disability worldwide. Lancet Neurol. (2023) 22:1096–8. doi: 10.1016/S1474-4422(23)00387-3 37827181

[2] Dziewas R Michou E Trapl-Grundschober M Lal A Arsava EM Bath PM . European stroke organisation and European Society for Swallowing Disorders guideline for the diagnosis and treatment of post-stroke dysphagia. Eur Stroke J. (2021) 6:LXXXIX–CXV. doi: 10.1177/23969873211039721, 34746431 PMC8564153

[3] Baik SM Kim M Lee JG. Comparison of early enteral nutrition versus early parenteral nutrition in critically ill patients: a systematic review and Meta-analysis. Nutrients. (2024) 17:10. doi: 10.3390/nu17010010 39796444 PMC11723109

[4] Barazzoni R Deutz NEP Biolo G Bischoff S Boirie Y Cederholm T . Carbohydrates and insulin resistance in clinical nutrition: recommendations from the ESPEN expert group. Clin Nutr. (2017) 36:355–63. doi: 10.1016/j.clnu.2016.09.010, 27686693

[5] Lin J Cai C Xie Y Yi L. Acute glycemic variability and mortality of patients with acute stroke: a meta-analysis. Diabetol Metab Syndr. (2022) 14:69. doi: 10.1186/s13098-022-00826-9, 35538585 PMC9092773

[6] Wu D Dai J Sheng Y Lin Y Ye H Wang D . Evidence summary on pain management in thoracoscopic lung cancer surgery. Asia-Pac J Oncol Nurs. (2025) 12:100693. doi: 10.1016/j.apjon.2025.100693, 40291140 PMC12022630

[7] DiCenso A Bayley L Haynes RB. Accessing pre-appraised evidence: fine-tuning the 5S model into a 6S model. Evid Based Nurs. (2009) 12:99.2–99.101. doi: 10.1136/ebn.12.4.99-b, 19779069

[8] Brouwers MC Kho ME Browman GP Burgers JS Cluzeau F Feder G . AGREE II: advancing guideline development, reporting and evaluation in health care. CMAJ. (2010) 182:E839–42. doi: 10.1503/cmaj.090449, 20603348 PMC3001530

[9] Shea BJ Reeves BC Wells G Thuku M Hamel C Moran J . AMSTAR 2: a critical appraisal tool for systematic reviews that include randomised or non-randomised studies of healthcare interventions, or both. BMJ. (2017) 358:j4008. doi: 10.1136/bmj.j4008, 28935701 PMC5833365

[10] Joanna Briggs Institute. JBI Critical Appraisal Tools. Adelaide: JBI.

[11] Foster MJ Shurtz S. Making the critical appraisal for summaries of evidence (CASE) for evidence-based medicine (EBM): critical appraisal of summaries of evidence. JMLA. (2013) 101:192–8. doi: 10.3163/1536-5050.101.3.008 23930089 PMC3738079

[12] Chen G Shen C Pan C Gao X Sun M Li X . Summary of best evidence for safe management of vasopressors through peripheral intravenous catheters. BMC Nurs. (2025) 24:1000. doi: 10.1186/s12912-025-03635-3 40745303 PMC12312446

[13] JBI Levels of Evidence and Grades of Recommendation Working Party. Supporting document for the JBI levels of evidence and grades of recommendation. Adelaide: JBI (2014).

[14] Wu B Peng B Wang YJ Wu SM Zhou LX Pan SY . Chinese guidelines for the management of severe stroke 2024. Chin J Neurol. (2024) 57:698–714. doi: 10.3760/cma.j.cn113694-20231024-00261

[15] Compher C Bingham AL McCall M Patel J Rice TW Braunschweig C . Guidelines for the provision of nutrition support therapy in the adult critically ill patient: the American society for parenteral and enteral nutrition. JPEN J Parenter Enteral Nutr. (2022) 46:12–41. doi: 10.1002/jpen.2267, 34784064

[16] Singer P Blaser AR Berger MM Alhazzani W Calder PC Casaer MP . ESPEN guideline on clinical nutrition in the intensive care unit. Clin Nutr. (2019) 38:48–79. doi: 10.1016/j.clnu.2018.08.037, 30348463

[17] Wunderle C Gomes F Schuetz P Stumpf F Austin P Ballesteros-Pomar MD . ESPEN guideline on nutritional support for polymorbid medical inpatients. Clin Nutr. (2023) 42:1545–68. doi: 10.1016/j.clnu.2023.06.023, 37478809

[18] National Health Commission of the People's Republic of China. Guidelines for the prevention and treatment of stroke in China (2021 edition). National Clinical Practice Guidelines Database. (2021). Available online at: https://guide.medlive.cn/guideline/24097 (accessed February 3, 2026).

[19] West China evidence-based nursing Center, Sichuan University, Nursing Management Committee, Chinese nursing association, neurosurgery branch, Chinese Medical Association. Chinese guidelines for enteral nutrition nursing in stroke patients. Chin J Evid Based Med. (2021) 21:628–41. doi: 10.7507/1672-2531.202101115

[20] Roberts AW Penfold S. Glycaemic management during the inpatient enteral feeding of people with stroke and diabetes. Diabet Med J Br Diabet Assoc. (2018) 35:1027–36. doi: 10.1111/dme.13678 30152589

[21] Joint British Diabetes Societies for Inpatient Care (JBDS-IP) Group. (2024). Glycaemic management during enteral feeding for people with diabetes in hospital: a guideline from the Joint British Diabetes Societies for Inpatient Care (JBDS-IP) group. JBDS-IP. Available online at: https://abcd.care/sites/default/files/resources/JBDS_05_Enteral_Feeding%20_Guideline_April_2024.pdf (accessed April 10, 2026).

[22] Honarmand K Sirimaturos M Hirshberg EL Srinivasan V Umpierrez GE Jacobi J . Society of Critical Care Medicine guidelines on glycemic control for critically ill children and adults 2024. Crit Care Med. (2024) 52:e161–81. doi: 10.1097/CCM.0000000000006174, 38240484

[23] Wu Z Liu J Zhang D Kang K Zuo X Xu Q . Expert consensus on the glycemic management of critically ill patients. J Intensive Med. (2022) 2:131–45. doi: 10.1016/j.jointm.2022.06.001 36789019 PMC9923981

[24] Rebollo-Pérez MI Florencio Ojeda L García-Luna PP Irles Rocamora JA Olveira G Lacalle Remigio JR . Standards for the use of enteral nutrition in patients with diabetes or stress Hyperglycaemia: expert consensus. Nutrients. (2023) 15:4976. doi: 10.3390/nu15234976 38068834 PMC10707756

[25] Heuschkel R Duggan C. "Enteral feeding: gastric versus post-pyloric". In: Connor RF, editor. UpToDate. Waltham, MA: Wolters Kluwer (2025).

[26] Oliveira-Filho J Mullen MT. "Initial assessment and management of acute stroke". In: Connor RF, editor. UpToDate. Waltham, MA: Wolters Kluwer

[27] Seres D Patel J. "Nutrition support in critically ill adult patients: parenteral nutrition". In: Connor RF, editor. UpToDate. Waltham, MA: Wolters Kluwer

[28] Xu HJ Wu LL Zhang Q. Best evidence summary for the management of intravenous insulin infusion in ICU patients. Chin J Nurs. (2023) 58:1489–95. doi: 10.3761/j.issn.0254-1769.2023.12.012

[29] Shi SS Ren HT Song LF Li JN Zhou Y Zhang Y. Best evidence summary for the management of hyperglycemia after enteral nutrition in patients with severe stroke. Clinical Focus. (2024) 39:1000–6. doi: 10.3969/j.issn.1004-583X.2024.11.007

[30] Wang W Qu H Chu J Meng DP. Best evidence summary on the management of hyperglycemia related to enteral nutrition in critically ill patients. Chin J Crit Care Nurs. (2022) 3:157–62. doi: 10.3761/j.issn.2096-7446.2022.02.012

[31] Hryciw BN Ghossein J Rochwerg B Meggison H Fernando SM Kyeremanteng K . Glycemic variability as a prognostic factor for mortality in patients with critical illness: a systematic review and meta-analysis. Crit Care Explor. (2024) 6:e1025. doi: 10.1097/CCE.0000000000001025, 38222872 PMC10786590

[32] Doola R Greer RM Hurford R Flatley C Forbes JM Todd AS . Glycaemic variability and its association with enteral and parenteral nutrition in critically ill ventilated patients. Clin Nutr. (2019) 38:1707–12. doi: 10.1016/j.clnu.2018.08.001, 30170779

[33] Sharma SK Rani R Thakur K. Effect of early versus delayed parenteral nutrition on the health outcomes of critically ill adults: a systematic review. J Crit Care Med. (2021) 7:160–9. doi: 10.2478/jccm-2021-0011, 34722919 PMC8519384

[34] Seifi N Amin Mohammadi M Dabagh AE Sedaghat A Rezvani R Khadem-Rezaiyan M . The effect of early enteral nutrition supplemented with synbiotics on lipid and glucose homeostasis in critically ill patients: a randomized controlled trial. Diabetes Metab Syndr. (2022) 16:102352. doi: 10.1016/j.dsx.2021.102352, 34972039

[35] Huang W Zhu L Song W Zhang M Teng L Wu M. Crosstalk between the gut and brain in ischemic stroke: mechanistic insights and therapeutic options. Mediat Inflamm. (2022) 11:6508046. doi: 10.1155/2022/6508046 PMC9578915 36267243

[36] Krishnan M Babu S Rahmathullah A. Dual dietary assault: unraveling the synergistic impact of high-fat and high-sucrose intake on type 2 diabetes pathogenesis. Mol Nutr Food Res. (2026) 70:e70346. doi: 10.1002/mnfr.70346, 41316994

[37] Belli M Bellia A Sergi D Barone L Lauro D Barillà F. Glucose variability: a new risk factor for cardiovascular disease. Acta Diabetol. (2023) 60:1291–9. doi: 10.1007/s00592-023-02097-w, 37341768 PMC10442283

[38] Ceriello A Monnier L Owens D. Glycaemic variability in diabetes: clinical and therapeutic implications. Lancet Diabetes Endocrinol. (2019) 7:221–30. doi: 10.1016/S2213-8587(18)30136-0 30115599

[39] Palaiodimou L Lioutas VA Lambadiari V Theodorou A Themistocleous M Aponte L . Glycemic variability of acute stroke patients and clinical outcomes: a continuous glucose monitoring study. Ther Adv Neurol Disord. (2021) 14:17562864211045876. doi: 10.1177/17562864211045876, 34589140 PMC8474316

[40] Di Bartolomeo AE Chapman MJ Zaknic AV Summers MJ Jones KL Nguyen NQ . Comparative effects on glucose absorption of intragastric and post-pyloric nutrient delivery in the critically ill. Crit Care. (2012) 16:R167. doi: 10.1186/cc11522 22985684 PMC3682265

[41] Rouhi AD Leon S Roberson JL Shreve LA Nadolski GJ Williams NN . Comparison of gastrostomy techniques in stroke patients with dysphagia: an entropy-balanced analysis. J Surg Res. (2024) 303:579–86. doi: 10.1016/j.jss.2024.09.064, 39437597

[42] Gosmanov AR Umpierrez GE. Management of hyperglycemia during enteral and parenteral nutrition therapy. Curr Diab Rep. (2013) 13:155–62. doi: 10.1007/s11892-012-0335-y, 23065369 PMC3746491

[43] Ohshiro Y. Continuous feeding insulin injection (CFII): a new simple method to stabilize severe glucose variability and nutrition delivery in critically ill patients. Cureus. 17:e78758. doi: 10.7759/cureus.78758 39931500 PMC11810147

[44] Hiemstra FW Stenvers DJ Kalsbeek A de Jonge E van Westerloo DJ Kervezee L. Daily variation in blood glucose levels during continuous enteral nutrition in patients on the intensive care unit: a retrospective observational study. EBioMedicine. (2024) 104:105169. doi: 10.1016/j.ebiom.2024.105169, 38821022 PMC11177052

